# Experiences of living with long COVID during childhood and adolescence: a qualitative study from the child’s perspective

**DOI:** 10.1186/s12887-025-06173-8

**Published:** 2025-10-03

**Authors:** Emmy Lillieberg, Per Ertzgaard, Eva Fernlund, Karel Duchen, Patrik Rytterström, Charlotte Angelhoff

**Affiliations:** 1 Department of Paediatrics in Jönköping, Barnkliniken, Länssjukhuset Ryhov, Region Jönköping County, Jönköping, 551 85 Sweden; 2 Department of Biomedical and Clinical Sciences, Linköping University, Linköping, Sweden; 3 Department of Rehabilitation Medicine in Linköping and Department of Health, Medicine and Caring Sciences, Linkoping University, Linköping, Sweden; 4 Department of Biomedical and Clinical Sciences, Crown Princess Victoria Children´s Hospital, Linköping University, Linköping, Sweden; 5 Linköping, Sweden; 6 Department of Biomedical and Clinical Sciences, Allergy Center in Linköping, Linköping University, Linköping, Sweden; 7 Department of Health, Medicine and Caring Sciences, Linköping University, Norrköping, Sweden; 8 Department of Health, Medicine and Caring Sciences, Allergy Center in Linköping, Linköping University, Linköping, Sweden

**Keywords:** Child, COVID 19, Health care, Long-term illness, Paediatrics, Post-acute COVID-19 syndrome, Post-COVID-19 among children

## Abstract

**Background:**

In February 2023, the World Health Organization (WHO) defined long COVID in children, highlighting limited knowledge about its psychosocial impact. Studies show it as a complex, long-lasting condition affecting multiple systems. WHO and researchers emphasise the need for more understanding, particularly its effect on daily life. The aim of this study was to explore how life is experienced and how it changed whilst living with long COVID during childhood.

**Methods:**

We present a qualitative study with an inductive and exploratory approach. Between October 2022 and March 2024, 16 children between 9 and 18 years old diagnosed with long COVID were interviewed face-to-face using a semi-structured interview guide. The results were analysed using reflexive thematic analysis by Braun and Clarke.

**Results:**

The results present the subjective reality of children suffering from long COVID and their struggle in daily life. The findings are presented through three themes: *Losing my foothold*, *Fatigue decides my path*, and *My way forward*, illustrating a temporal and emotional journey, reflecting how children make sense of their experiences, adapt to the persistent impact of long COVID, and gradually move toward acceptance.

**Conclusions:**

This study addresses the lack of knowledge of long COVID in the society, how it affects children in their struggle to find a new path in life. It also shows that, with knowledge and support, the symptoms and the burden of the condition can decrease or even pass. It is important that people around these children, including health care, school and family, use this knowledge to promote health and avoid educational, health and social problems at a vulnerable time in life.

**Supplementary Information:**

The online version contains supplementary material available at 10.1186/s12887-025-06173-8.

## Background

When COVID-19 infection initially appeared in China in December 2019, the global population was unaware of the significant impact it would have on life in the subsequent years. Now, the world has adapted to the presence of Sars-Cov-2 virus. However, over the years, we have increasingly learned of children experiencing long-term symptoms following a COVID-19 infection [1].

Long-term symptoms after a severe COVID-19 infection occurs for all ages in at least 10% of infected individuals, with more than 200 symptoms identified that impact multiple organ systems [2]. Children may develop post-infectious symptoms with year-long durations, independent of initial severity of the COVID-19 infection [3]. Such symptoms have been referred to as post-acute sequelae of COVID-19 (PASC), long COVID or post-COVID-19 condition [2, 4]. In this paper, we use the WHO definition of post COVID-19 in children and adolescents, but refer to it using the more common term “long COVID”.

Consensus was lacking about the definition and diagnosis of long COVID among young people until February 2023, when WHO established criteria for long COVID in children and adolescents. Post COVID-19 condition in children and adolescents occurs when the individual has a history of confirmed or probable COVID-19 infection. Symptoms of long COVID should last at least two months and occur within three months after an acute COVID-19 infection and should impact everyday life. A single symptom is sufficient if such an impact is substantial. Symptoms can persist from the acute infection, but may also be new after initial recovery, and can fluctuate or relapse over time [4].

A national study of long COVID in children in England – the CLoCk study – reported that 66% of children (*n* = 2038) who tested positive for SARS-CoV-2 virus continued to experience symptoms at a three-month follow-up, 30.3% (*n* = 928) of whom presented with three or more symptoms [3]. In Sweden, a study of 55 children admitted to a paediatric hospital in 2020 found that 21% (*n* = 12) had persistent symptoms, with fatigue being the most commonly reported [5]. Similarly, Mogensen et al. examined long COVID symptoms in young adults (*n* = 2022, mean age 26.5 years) from the Swedish population-based BAMSE birth cohort. Participants had completed repeated questionnaires on COVID-19 symptoms, which showed that 16.5% reported symptoms consistent with long COVID [6]. In the United States, a large-scale study involving 5 376 children found that 36% reported symptoms affecting multiple organ systems, with the prevalence and type of symptoms varying by age group. Younger children differed from adults in symptoms, which generated a higher risk of missing the diagnosis and possible treatment [7].

Even if the symptoms are not life threatening, they can have an impact not only for the individual and family, but also on public health due to their high prevalence and long-term symptoms [6]. The symptoms affect the children or adolescents to different extents, with some experiencing significant impacts that require them to adapt to a new reality [8]. Buonsenso et al. showed that in several cases children with symptoms after a COVID-19 infection had such severe manifestations that they were unable to take part in normal activities such as school, sport or leisure activities, which they had participated in before the infection [9]. This can affect self-esteem and identity, and may lead to the experience of social exclusion and isolation, as shown in adults with long COVID in several studies [10, 11]. However, only a few studies have asked the children or adolescents what it is like to live with long COVID. In the UK, four female teenagers aged 10–17 years were interviewed about their experiences of living with long COVID [12]. The results showed that their symptoms affected their lives in several areas, for example school, friends and family. Angelhoff et al. explored the challenges parents faced when living with a child suffering from long COVID, including feeling distrusted and dismissed when seeking medical care, and often being told the symptoms were psychological or psychosomatic. Their study also highlighted the significant impact of long COVID on family life, with changes in routines, social activities, and the home environment, as well as parents and siblings having to set aside their own needs to support the child [13].

WHO have declared the need for more knowledge about long COVID among children and adolescents [4]. Al-Aly et al. also highlights this, stating that more research is required on how it affects the individual’s life, which social responses it generates, and the perception of possible social stigma [14]. Research focusing on adults cannot be readily generalized to children and adolescents, as their experiences and needs may differ significantly. As noted by Field (2004), it is essential to include children in research to ensure they have the opportunity to fully benefit from medical advancements. Therefore, engaging children and adolescents in studies related to COVID-19 is crucial for understanding and addressing their specific challenges [15]. In the United States, when the de Beaumont Foundation asked physicians about their knowledge of long COVID, it found that 78% agreed that long COVID is a problem, but only 25% felt prepared to address it [16]. This emphasises the need for more knowledge and information. To truly understand how long COVID affects children and adolescents, it is essential to listen to their own voices and stories. A qualitative approach makes it possible to capture their unique perspectives and lived experiences, which might otherwise remain unheard. Thus, the aim of this study was to explore what life is like for children and adolescents living with long COVID, to describe the challenges they are confronted with and how their life has changed.

## Methods

### Design

We conducted a qualitative interview study with an inductive and exploratory approach. We used constructionist epistemology throughout our analysis, following reflexive thematic analysis by Braun and Clarke [17–19], and the principles of the Reflexive Thematic Analysis Reporting Guidelines, RTARG [20]. Our approach was inductive, experiential and exploratory, incorporating a hermeneutic perspective on data interpretation [17–20]. The study was part of the POCOKIDS (*Post COVID in Kids*) study, which is a larger multiprofessional research project [21]. The aim of the latter is to examine, describe and understand physiological, psychological and social effects for not only the child but also the family.

### Participants and procedure

The study group in the POCOKIDS study comprises 29 children long COVID. Inclusion criteria were age between 6 and 18 years with a diagnosed long COVID referred to paediatric clinics in the counties of Östergötland and Jönköping in Sweden. First, the participants were invited to the paediatric clinic for medical screening by a paediatrician to exclude other differential diagnoses. After oral information was given, a written informed consent was obtained from the study participants over 15 years of age, or from the legal guardians of younger participants.

After informed consent was obtained, we used purposeful sampling to recruit children with variation in gender, age, and municipality, in order to capture a broad range of experiences relevant to the study aim. Oral and written information about the study was given during the first medical examination. A parent of the child was contacted later by phone (CA or EL) to decide the time and place for an interview. The participants were encouraged to choose a time and place that suited them to ensure that they would be relaxed and undisturbed during their interview.

A semi-structured interview guide about the child’s symptoms and changes in health since their COVID-19 infection was constructed by two of the authors (CA and PR) for this project and was used as a support during the interview. See Supplementary file for full interview guide.

### Data collection

Seventeen children and adolescents, 10 girls and 7 boys, from five different municipalities in the southeast of Sweden agreed to participate. However, one child was unable to take part due to their own health condition. In total, 16 interviews were conducted. Median age was 15 years (range 9–18 years). The children’s first COVID-19 diagnosis was confirmed between March 2020 and April 2022. Half of the group (*n* = 9) contracted their first COVID-19 infection between March and December 2020. Interviews were conducted between October 2022 and March 2024, 7–42 months after the child’s primary COVID-19 infection, see Table 1.

**Table 1:** Participants

Fictional name	Gender	Age	Months since first COVID-19 infection at date of interview
Julie	Female	17	34
Karl	Male	14	30
Agnes	Female	18	31
Natalie	Female	17	27
Johanna*	Female	14	17
Elsa	Female	18	24
Paul*	Male	9	31
David	Male	13	31
Malin	Female	17	21
Anna*	Female	10	7
Fanny	Female	18	33
John	Male	16	42
Samuel	Male	14	42
Elena*	Female	13	22
Christian	Male	18	22
Olivia*	Female	10	22

* = a parent accompanied the children during interview

Nine interviews took place in the children’s homes, and seven in meeting rooms at paediatric clinics. A pilot interview was conducted to ensure that the interview guide was appropriate and comprehensible. As no revisions were deemed necessary following this interview, it was included in the final analysis. The interviews were conducted face-to-face by CA (registered paediatric nurse with substantial experience in qualitative research and in interviewing children and adolescents) and EL (PhD student and resident paediatrician with extensive experience working with children and adolescents).There was no previous clinical or personal contact between the interviewer and the children. During the interview, an informal conversation took place for a few minutes for the interviewer and child to get to know each other. As the interviews were with children of different ages, the following aspects were pointed out before the interviews took place: the children were given age-adapted oral information about the study as well as an opportunity to ask questions before the recording started. They were also informed that there were no right or wrong answers. This was to ensure a child-centred process and that the children felt that they could express themselves freely. A parent was allowed to be present during the interview if requested by the child, but was asked to remain in the background to allow the child to speak freely. In five of the interviews, a parent was seated beside the child. In two of these, the parent shared their own experiences and perspectives during the interview, despite the interviewer’s efforts to direct the conversation towards the child. The children were encouraged to tell their story and to give examples and descriptions about their experience. During the interviews, we adjusted the questions depending on the child’s age, development and comorbidity. Moreover, we sometimes worked with shared thinking to help them express their feelings and thoughts [22]. If the children expressed thoughts and feelings during the interview that needed support and assistance from health care, we had pre-processed routines for this. One such routine was used as a participant described experiencing poor mental health due to their condition.

All interviews were audio recorded and transcribed verbatim. The recorded interviews had a median time of 28 min. In the result, we use fictional names to ensure anonymity while retaining the personal impression.

### Data analysis

Data analysis was conducted after all interviews had been completed. The interviews were transcribed verbatim. First, familiarisation with the data was achieved through repetitive listening and reading of all the interviews, notes were taken if a special thought or feeling came up. After that, meaningful codes were extracted from each interview and organised in different groups. The reading, quotes and codes were discussed by a group of three researchers (EL, CA, PR), to check the credibility of the coding. The work progressed successively, moving from codes back to each interview to detect common meaning and to create categories and themes that reflected the core meaning of the interviews. During the process, we continuously varied individual work with close cooperation in the group. We rechecked the relationship between the data and the themes, reading all codes and the entire data to confirm thematic consistency. Finally, after several rounds of reading and processing the result with all authors, the definitions and names of themes were set. See example Table 2.

**Table 2:** Examples from the data analysis process: quotes, codes, subthemes, and themes

Quotes	Codes	Subtheme	Theme
…this has come along so slowly, so to speak, so I don’t know if I have more things that… well, this just feels like the new normal now, sort of.	Developed slowly, do not know if there is more things, becoming the new normal	Seeking answers where there are none	Losing my foothold
But I remember saying, like, yeah, we don’t need to talk about it or tell anyone. Yeah. It wasn’t like that the second time. I don’t think… no, I don’t want that now either. I don’t want it to be like it’s written all over my face—post-Covid—I don’t want that. I guess that’s maybe what I was a bit afraid of.	Do not want to be like post-Covid written all over the face, afraid of it	Disrupted lives	
No, it can be anyone really—relatives, family members… I know my dad had a hard time understanding. He thought I was just making it up, like, “Oh, she’s just pretending, she’s not actually feeling bad,” and so on. Yeah, so it can be a lot, really…	Relatives, family members for example father difficulty understanding, believed I was exaggregating	Support and understanding	

## Results

**Fig. 1:**
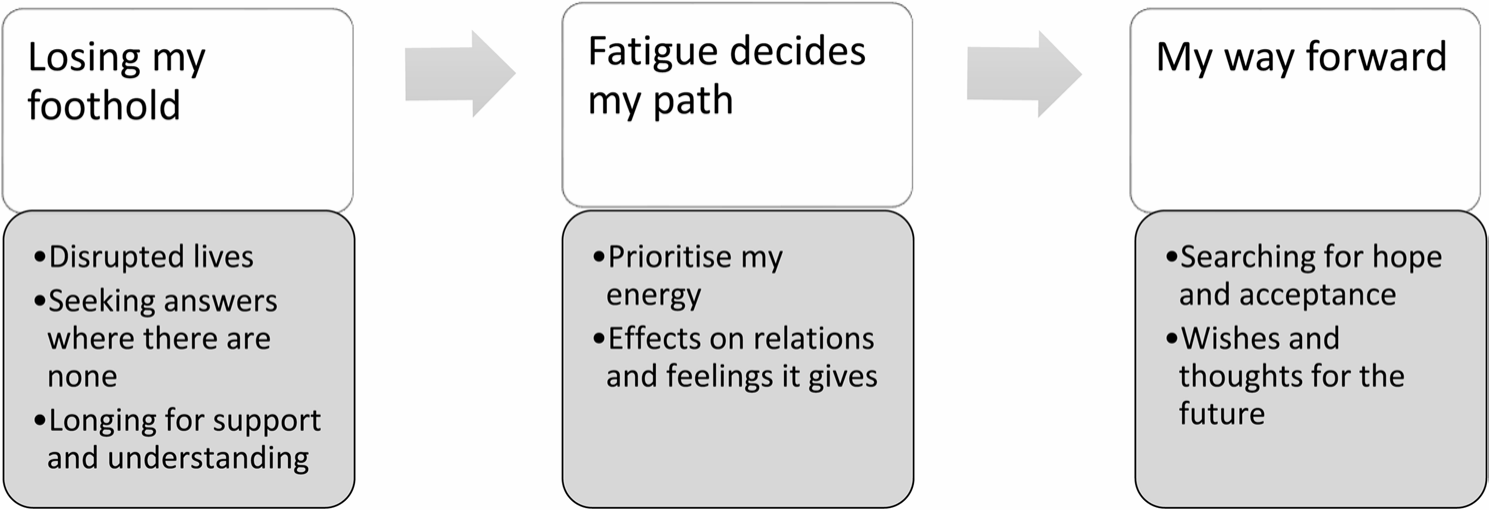
Themes and subthemes

Three overarching themes emerged from the interviews: (1) Losing my foothold, (2) Fatigue decides my path and (3) My way forward. The themes can be understood as unfolding a timeline that reflects the child’s journey through making sense of their experiences, living with, and attempting to accept, the reality of long COVID, see Figure one. Seven subthemes are displayed in Fig. 1 and are presented in separate sections in the result.

### Losing my foothold

This theme captures the experiences of realising that something was wrong, the impact of various symptoms on their daily lives, their efforts to seek help, and the emotions associated with that search.

#### Disrupted lives

The subtheme “disrupted lives” describes the first time the children fall ill and how aspects of everyday life that they took for granted are now transformed into something questionable that they do not recognise. The beginning can be different. For some children, the disruption in everyday life comes to mind successively, with thoughts on what was happening, whereas others had a sudden start. Regardless of whether the symptoms developed quickly or gradually, children feel that there lives are disrupted, and the path that was once so familiar is now unrecognisable. They are forced to find a new path in life. Samuel, 14 years old, described it as “*COVID-19 knocked out my whole body”*, which hospitalised him for months. Even two years after the acute infection, he can only take a few steps by himself, and is still dependent on crutches and a wheelchair. He cannot cycle nor can he walk longer than five to ten metres by himself. These are limitations compared with the life he wishes to live, such as going for walks or bike rides together with his friends. Samuel’s experience also reflects a feeling of loneliness. When the children are no longer able to do the activities they used to be able to do, they also lose the context in which they spend time with other friends. For example, Christian, 18 years old, explains what happens if he tries to exercise: “*leaving me with fever*,* sore throat and completely exhausted days after”*. He ends up not exercising and not being able take part in what has been an important part of his life. Exercising and the wealth that comes with it, but also being part of a context and a group, are important for the children. When these are taken away from them, it leaves them facing a new reality with less coherence in life and the loss of many of their friends.

Another way of losing foothold is when they experience symptoms of tiredness, and autonomic symptoms such as a rushing pulse. For Fanny, 18 years old, it has the following effect:

Showering…//… I sit on a chair in the shower because otherwise I’d faint, and that’s not fun either… eighteen years old and have a chair in the shower.

#### Seeking answers where there are none

The symptoms raise thoughts about what is normal, within the person itself but also among parents, school and even the health care. The children ask themselves why they can no longer walk the way they have done before. This uncertainty is reinforced by the answers of those around them. When they try to seek answers, the children first reach out to their parents for support. This could be easy at times, but the answers could also meet a lack of understanding, as in the case of Elena, 13 years old, when she reached out to her father:

I know my dad had a hard time understanding. He thought I was just making it up, like, ‘Oh, she’s making it up, she’s not really feeling bad,’ and so on.

When reaching out to health care, they are met by answers such as: “no blood tests or other investigations showed that anything was wrong, the symptoms are normal at your age”. John, 16 years old, describes his experience of his journey with health care, starting when he was 14 years old; “*during the whole period of sickness*,* there has been a lack of knowledge*,* only examining me and not treating me”*. This in turn causes the children to question their own feelings and symptoms, isolating them and their families with a sense of not feeling well and feeling helpless. John expressed it as: “*if the health care does not help*,* what can I do then?”*

#### Longing for support and understanding

The children are trying to find a new way forward by searching and longing for understanding. Yet, they have different experiences. Even if information and knowledge about long COVID have been sparse, children describe genuine support from people around them, such as friends and family. Karl, 14 years old, shared that his friends are always happy to see him when he returns to school after being absent due to long COVID symptoms. They also try to support him in managing schoolwork. However, both he and others expressed feeling unsupported and left alone with their thoughts about the future. Karl described a deep sense of uncertainty, wondering, *“Will I have this sickness for the rest of my life?”*, a question that reveals his longing for someone to share his thoughts with. The children expressed feelings of loneliness, a strong need for someone to talk to, yet they often had no one to confide in. At times, they had even lost hope of being truly understood. John, on the other hand, did not want to talk with anyone;

…//… I already felt so left out, you know. Eh… when I couldn’t be part of things and do stuff, so it was like…//… I thought it would just get worse if I told them this too. You don’t want to be even more different, you know.

School plays a central part in the child´s perception of support or lack of support. For example, Christian, 18 years old, experienced support from the school. The school and health care worked together to ensure that the knowledge and support available would be the best possible for him as he tried to find his new way. For example, they reduced time in school to half a day in the beginning and minimised physical activity as this made him feverish and exhausted for several days. Nevertheless, nearly every child describes some parts of their everyday life where it has been hard to receive support and understanding. David, a 13 year old, tells us about his reality:

.//… we’ve got some support now with these video meetings, but before that, they didn’t even want to show me what I needed to know for exams and how I should do it. Some have even gone as far as to say that it’s not their job to help me with schoolwork.

### Fatigue decides my path

This theme focuses on experiences of fatigue, how it can express itself and what consequences it has physically, psychologically and socially for the children. Fatigue has been a central part in all the children’s description of their everyday life. The children express fatigue in words like lack or loss of energy, or tiredness. Paul, a 9 year old, said:

Yeah… tired, tired all over my body…//…Tired to the point where you kind of don’t have the energy for anything…//…And… in my brain too.

#### Prioritise my energy

Because of fatigue the children need to prioritise their energy. An important part of the children´s life is school, where their fatigue clearly decides their path even if they do not want it to. All the children repeatedly come back to the importance of school and how they need to struggle to manage it. Even if the children manage to come to school, the time in school is not the same as before. John, 16 years old, said that even if he was there, he only talked with others about what was given for the schoolwork; *“my energy was not enough to socialise even if I wanted to.”* Long COVID also comes with absence from school. Some children were not at school for weeks, some for years. Others need to have a personal schedule because they lack the capacity to be in school a whole day. After school, the children describe a need to lie down on their bed and rest, some for an hour and some for the rest of the day.

Because of fatigue, they start to prioritise their energy expenditure. First, they start to minimise activities during weekends, just to get the rest they need to manage school. John expressed it as;

Eh, stay at home, just resting…//…eventually, I became completely convinced that that’s what you’re supposed to do at weekends…//…if I’m going to have enough energy to handle next week, I have to stay at home now and rest. And of course, I shouldn’t go along my family and what they’re doing, because I need to rest. It just became natural in a way.

The children long to be physically active, but often choose not to be because of fatigue and the need to prioritise their energy expenditure. Elsa, 18 years old, tried to continue exercising but afterwards she needed to rest for two days, so she decided to stop; “*It was not worth the energy it took*.” David, 13 years old, had to quit table tennis, leaving him with a newly bought five-star racket, a bad feeling, and an emptiness in his life.

#### Effects on relationships and the feelings it gives

Another side effect of how fatigue determines the children’s path is its effect on relationships. Realising that they will be unable to fulfil goals and wishes affects the children and their entire whole family. The children express how their parents need to be off work to drive them back and forth from school, help them with homework, and contact school and health care. In addition, seeing their parents sad or worried on their behalf makes the children feel guilty about not being able to reach parental expectations. Christian, 18 years old:

Mum and dad…//… they probably think it’s quite tough because I can’t manage what I used to do, they’ve said, and I can see it in them…//… I saw that they were feeling really bad…//… it also made me feel like I… wanted to go away and show that I can, and stuff.

Because of the limitations long COVID came with, some children started online gaming. This afforded an opportunity to meet and associate with friends, to not completely lose contact with their social life. For Karl, 14 years old, online gaming gives him an opportunity to evade the harshness of reality.

.//… what makes me happy is that I can have a life online when I play games with my friends. …//… it’s like a different life, it’s better there…//… my thoughts are better, I don’t think about my real life, I feel better in my gaming life…//… games make me happy because they take me away from my life by letting me live another life.

The reduced ability to take part in different social activities because of fatigue comes at a price. It affects relationships with others, both family and friends. Elena, 13 years old, looks back and faces the reality that she sees two friends only seldom now. The friends she had before are not there anymore because, as she puts it: “*they got tired of me not being able to do so much anymore*.” Social activities demand a need for rest afterwards, sometimes for several days. Paul, 9 years old, tells us about what he wishes for but cannot do anymore:

Eh… be with friends a lot, quite a bit. Be with friends, like…//… Because I can’t manage. If I can manage that day, the day after I get really, really tired and can barely do anything at all.

### My way forward

Following a timeline, this theme focuses on how time has evolved and the children’s thoughts on their coming years ahead. The children describe their way forward; the symptoms are improving for some, and some even feel themselves again. However, they also address the difficulty of remembering what it was like before, as some have had long COVID for several years, and it has become their normal state. As they successively get better, they manage to do things that give them energy and joy, such as exercising, being with friends, and being at school.

#### Searching for hope and acceptance

One way they express their way forward is through hope and acceptance. The feeling of improvement and success, showing themselves and people around them that things can be better, gives hope. Not only by thinking it but also by hearing it from people around them, especially from health workers working with long COVID, that it will get better gives the children peace and hope for the future. Julie, 17 years old, tells us about her new motto in life, which she got from a psychologist:

Well, life is like a card game…//… you get a hand and then you simply have to make the best of it.

Acceptance was an important part of finding a way forward. Natalie, 17 years old, expresses her frustration at not being able to play football as she refuses to accept that she cannot do it. Her wish is to be able to just kick the ball and play together with her family, but she doubts if she is ever going to be able to do it. In contrast, John, 16 years old, tells us about part of his journey forward:

…//… when I was sick, when I was like, at my sickest, I didn’t really have any kind of thoughts about the future, you know… I also talked to a psychologist about it. And that was a big thing, like, being able to feel some hope for the future. Because when I felt the worst, I thought that it wasn’t at all certain that I would survive this period of illness. So, there was really no reason for me to think about the future, like, what would I do in the future, you know, really.

One of the biggest realisations for John has been to understand what feeling bad is like and how it can make everything feel completely hopeless, even when it is not. In addition, things can get better, even if it does not always seem that way. For John, accepting his illness has been the hardest, but also most important, part. Questions like *Why me?* have spun endlessly around his head. Coming to terms with the fact that he is sick and that his life has changed has been a long and difficult process. However, now he expresses an acceptance and satisfaction with his life, however limited it may be, which is helping him move forward.

#### Wishes and thoughts for the future

Christian, 18 years old, expresses a wish not to have long COVID. However, even if the wish exists, he accepts his sickness and its limitations, and is starting to see himself as a normal person, which he did not do before he reached acceptance. He also has a confidence in his body, that it will resolve the issue; “*sometime in the future I will be okay.”* In contrast to John and Christian, other children expressing sadness and resignation, and did not dare to dream. Not knowing if he/she is ever going to be well again, still trying to live their lives anyway. Natalie, 17 years old, says:

I don’t really dare to have dreams anymore, now that I have long COVID…//… my first goal or dream is to get better.

Wishes for the future on their way forward do not need to be huge. Natalie, 17 years old, says that she wishes to; “*not need to plan what I am doing and not having to worry that my energy is not sufficient*,* that I can go out with my friends and not need to rest the next day.”* Another example, Samuel, 14 years old, wishes to have the energy to be with his friends, play outside and to manage to cycle again. Or, as Elena, 13 years old puts it: “*I just want to feel better*.”

A wish the children express is to be able to go back to school. To manage to complete their education together with their friends. Karl, 14 years old, tells us about how he wanted to be on “well-being leave”, not sick leave. To be off because he was well, to do something fun, instead of being off because his body couldn’t manage. He was off, but it was a negative kind of leave. It wasn’t fun to be off when he was supposed to be at school, when he was supposed to be with the other children, doing what everyone else was doing.

In the end, Fanny, 18 years old, reflected on her way forward and expressed a strong desire to contribute to something meaningful. Although she acknowledged that the research might not lead to any immediate help for her own situation, she emphasised how important it felt to be involved. For her, participating in the study was a way to make a difference – for herself, but even more so for others. She wanted to share her experiences to support further research and help ensure that other children with long COVID are seen, heard, and better understood. Having lived with symptoms since she was 15, and now, 18 and no longer attending school or engaged in daily activities, she expressed how vital it is that something is done to change the situation for the many young people affected.

## Discussion

This study aimed to explore how life is experienced and how it changes when living with long COVID during childhood. The findings are presented through three themes: *Losing my foothold*, *Fatigue decides my path*, and *My way forward*. These themes illustrate a temporal and emotional journey, reflecting how children make sense of their experiences, adapt to the persistent impact of long COVID, and gradually move toward acceptance. In line with reflexive thematic analysis, the results are interpreted as constructed patterns of meaning that remain relevant and present in the children’s narratives.

The current study shed light on how children feel alone and ignored when forced to find a new way in life because of long COVID and the symptoms that accompany it. Fatigue takes over a crucial part of their lives and contributes to their losing their former context, which studies among adults have also shown [23, 24]. Even if support from family, school and friends was present, there was a feeling of being alone, not knowing what is normal or not, with thoughts and worries about their future. The children have had to change their way of life, some to a great extent, which has affected not only them but also the people and the context around them. However, with support, belief and understanding, the children develop acceptance and, over time, the burden of symptoms can decrease.

Knowledge and research on long COVID among children and adolescents that focuses on the individual’s own experience and perspective are sparse. Only a few studies have been performed [12, 25]. Our results reflect a reality from children over a broad spectrum of ages, between 9 and 18 years old. Faux-Nightingale et al. point out the importance of being listened to and understood by people and the system around them when seeking knowledge about, and care for, a wide range of symptoms [12]. This affects a young person’s entire life, including school and family, as is evident in our study. The study conducted by Faux-Nightingale et al. compromises four interviews, all with females, and whose parents were included, and took an active part in, three interviews [12]. Messiah et al. held telephone interviews with parents of 25 children who had been hospitalised for COVID-19, where some suffered from symptoms long after their infection [25]. They illustrate a wide range of psychological symptoms, mostly focusing on the acute infection, but also highlight the side effects of the acute infection and exacerbation after a reinfection [25]. Our study emphasises the children’s own perspective in their own words. It includes children who were hospitalised and those who were not during their acute infection, affording a broader perspective on what it is like to live with long COVID.

A striking finding in the analysis were the fatigue experienced by the children and how severely it influenced the children. Schlegtendal et al. found that children with a previous Covid-19 Omicron infection displayed more cognitive and mental problems, and impaired physical performance than children without previous infection did [26]. The children in our study also reported a life strewn with impaired physical performance, leaving them unable to do what they had done before, and forcing them to face a new reality, namely an everyday life with the limitations that come with fatigue.

Although long COVID has clearly disrupted the children’s ability to live their lives as they normally would, they still find themselves having to fight for credibility and acceptance in the various contexts that surround them. They highlight the importance of trust and belief when they need to reach out to health care, for example, which parents also indicate as being important [13]. Wild et al. show that children with long COVID struggle to be understood due to a lack of diagnostic tests and the indiscernible nature of their symptoms. They also describe three dominant narratives – that COVID-19 is mild, that children are rarely affected, and that the pandemic is over – which conflict with the children’s experiences and hinder support, both in health care and in schools [27]. This is also displayed in our study, in which the children have to fight to have their symptoms understood in their own family, in health care and in school. It is a child’s right to be given the help and adjustments they need in school, according to the UN Convention on the Rights of the Child [28]. Failure to achieve their goals at school will affect their lives for many years to come. Some children in the present study report a reality in which they are rejected and not listened to when they reach out for help and adjustments that would enable them to manage at school.

One notable strength of this study is the decision to interview children and adolescents directly, allowing their own voices and perspectives to be heard regarding how long COVID has affected their lives. This approach aligns with the ethical imperative to include children in research that concerns them [15]. Another strength was the involvement of children and their families in choosing the location for the interview. This contributed to creating a safe and secure space, which helped the children express their own thoughts. This approach is consistent with Darcy et al., who emphasize the importance of context in accessing the lived experiences of children with illness [29]. The results support the need for a holistic understanding of what it is like to live with long COVID during childhood, particularly the importance of listening to the child’s subjective experiences. Our research group is multidisciplinary, representing a range of prior knowledge and approaches to the data. This diversity contributed to a broader and more nuanced interpretation of the findings.

However, the study also had limitations. In two interviews, parents participated more than intended—sometimes to support the child, but occasionally to share their own experiences. While parents’ perspectives are valuable, their involvement may have influenced the child’s ability to fully express their own views. Additionally, one child who wished to participate was unable to do so due to their current health status related to long COVID. This may have resulted in the loss of valuable data from a child whose illness had a significant impact on daily life.

The findings of this study can contribute to improvements in child-centred care for those affected by long COVID, and could also inform practices in other parts of society, such as schools, to help prevent social and educational difficulties during a particularly sensitive phase of life. Among the findings of the present study are the importance of being believed when reaching out for help; having adjustments made in the school environment, for example, when the fatigue becomes too great; helping the children gain acceptance; and giving them hope that the symptoms will decrease or even pass. The present study indicates that listening to children and seeing them as active collaborators, as well as developing individual goals and treatment plans in close collaboration with the children and their family, are cornerstones in child-centred care [30].

Future research could further explore how child-centred care can be adapted to meet the needs of children living with long COVID, particularly in school and healthcare settings. Longitudinal studies may be valuable to understand how children’s experiences and coping strategies evolve over time. Additionally, research involving larger and more diverse samples could help identify structural barriers and facilitators to support and recovery.

## Conclusions

The present study has shed light on a growing group of children who are suffering from long COVID about whom the knowledge of psychosocial effects on their daily life has been sparse. The children are struggling to be believed, both in their own family, in school and in health care. They are trying to manage fatigue, which claims a considerable amount of their time. However, with time, support and trust from people around them, their sickness can be accepted, and their quality of life can improve. The burden of symptoms can decrease or even pass, which the children highlight in the present study. To help the group of children suffering from long COVID to recover, it is important to meet them without bias, listen to them, and strengthen them in their way forward.

## Supplementary Information

Supplementary Material 1.

## Data Availability

The datasets analyzed during the current study are not publicly available due to ethical considerations and the need to protect the privacy of the participating children. However, the data are available from the corresponding author upon reasonable request.
