# Supplementary material for: Experiences of living with long COVID during childhood and adolescence: a qualitative study from the child’s perspective
Source: BMC Pediatr. 2025 Oct 3;25:754. doi: 10.1186/s12887-025-06173-8 (PMC12492641; doi:10.1186/s12887-025-06173-8)
Supplement: Supplementary file 1 — Supplementary Material 1. [file 12887_2025_6173_MOESM1_ESM.docx]

**
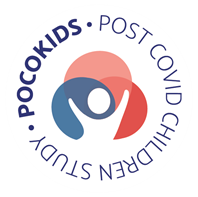
Interview guide**

1. What was it like when you had Covid?
2. What symptoms did you have?
3. What did you find the most difficult?
4. What was it like at home while you were sick?
5. Were there others in your family who were also sick?
6. Can you tell me how you’re feeling today? Symptoms?
7. How do you feel now compared to before you became ill?
8. Can you describe what a typical day looks like for you?
9. Can you describe your school situation today?
10. What do you usually do in your free time? Has it changed?
11. Can you describe your relationship with friends? Has it changed?
